# A Novel Tri-Axial Piezoelectric MEMS Accelerometer with Folded Beams

**DOI:** 10.3390/s21020453

**Published:** 2021-01-11

**Authors:** Yan Liu, Bohao Hu, Yao Cai, Wenjuan Liu, Alexander Tovstopyat, Chengliang Sun

**Affiliations:** The Institute of Technological Sciences, Wuhan University, Wuhan 430072, China; liuyan92@whu.edu.cn (Y.L.); hubohao@whu.edu.cn (B.H.); caiyao999@whu.edu.cn (Y.C.); lwjwhu@whu.edu.cn (W.L.); alxtov@whu.edu.cn (A.T.)

**Keywords:** piezoelectric accelerometer, MEMS, AlN/ScAlN thin film, sensitivity

## Abstract

Microelectromechanical (MEMS) piezoelectric accelerometers are diversely used in consumer electronics and handheld devices due to their low power consumption as well as simple reading circuit and good dynamic performance. In this paper, a tri-axial piezoelectric accelerometer with folded beams is presented. The four beam suspensions are located at two sides of the mass aligned with edges of the mass, and the thickness of the beams is the same as the thickness of the mass block. In order to realize the multi-axis detection, a total of 16 sensing elements are distributed at the end of the folded beams. The structural deformations, stress distribution, and output characteristics due to the acceleration in *x-*, *y-*, and *z*-axis directions are theoretically analyzed and simulated. The proposed accelerometer is fabricated by MEMS processes to form Mo/AlN/ScAlN/Mo piezoelectric stacks as the sensing layer. Experiments show that the charge sensitivity along the *x-*, *y-*, and *z*-axes could reach up to ~1.07 pC/g, ~0.66 pC/g, and ~3.35 pC/g. The new structure can provide inspiration for the design of tri-axial piezoelectric accelerometers with great sensitivity and linearity.

## 1. Introduction

Microelectromechanical (MEMS) accelerometers, known as the key components of the inertial measurement unit (IMU), are widely used in many applications, such as environmental monitoring, wearable devices, and mechanical vibration measurement [1]. Compared with piezoresistive and capacitive accelerometers, piezoelectric material-based accelerometers have the advantages of the wide operating frequency range, great linear characteristics, and high electromechanical coupling coefficiency [2,3].

The most commonly used materials for piezoelectric accelerometers are zinc oxide (ZnO) [4,5], aluminum nitride (AlN) [6,7], and lead zirconate titanate (PZT) [3,8]. Piezoelectric AlN-based accelerometer sensors offer advantages such as compatibility with a standard CMOS process, low dielectric constant, great piezoelectric coefficients, and mechanical properties [7,9]. F. Gerfers et al. [10] designed a novel accelerometer structure based on AlN thin film, which consists of tapered-beams with four balanced bars, and the tested charge sensitivity is 5.2 pC/g with low off-axis sensitivity. Doping Sc element into AlN is an effective way to enhance the value of piezoelectric strain coefficient, and studies found that ScAlN has a five times higher piezoelectric constant than pure AlN [11,12]. P.M. Mayrhofer et al. [13] fabricated and evaluated the performance of ScAlN and pure AlN-based energy harvesters. Experimentation showed that the increase of *e*_31_ of ScAlN film contributes to the improved output characteristics compared with AlN thin film.

The cantilever-based structure is the most popular design of piezoelectric accelerometers due to the easy fabrication process and high energy conversion efficiency [8,14]. A lot of research focuses on optimizing the structure of the cantilever beam to achieve high sensitivity, broad frequency bandwidth, low transverse coupling [15,16,17,18]. Don L. DeVoe [19] found that the sensitivity of the cantilever-based accelerometer with discrete masses is higher than that of the single cantilever-based accelerometer due to concentrated end load. Qiang Zou et al. [20] designed tri-axis piezoelectric-bimorph accelerometers with traditional cross-shaped beams. The unamplified sensitivity of the tri-axial accelerometer showed the sensitivity of 0.93, 1.13, and 0.88 mV/g in the *x*, *y*, and *z*–axes. Ma-hui XU et al. [21] presented a new type of tri-axial piezoelectric accelerometer with X-shaped cantilever beams. Simulation results showed that the charge sensitivity of the new X-shaped device is higher than the traditional cross-shaped accelerometer. In order to reduce the transverse interference of the uni-axis accelerometer, Bian Tian et al. [22] proposed a piezoelectric accelerometer with added beams. Except for the straight cantilever structure, Hui Yang et al. [23] found that the folded cantilever beams can improve the sensitivity of the accelerometer.

In spite of the many studies conducted towards increasing the sensitivity of accelerometers, piezoelectric accelerometers based on thin four beam suspension, especially tri-axial accelerometers, have not yet exhibited sufficient accuracy and stability for acceleration measurement over a wide frequency. One of the reasons is that the traditional cross-shaped accelerometer with four thin cantilever beams is more prone to unpredictable distortion under external loads, which leads to greater variations per specific changes applied in the measured parameter. In this work, a piezoelectric accelerometer comprised of multiple folded suspension beams is studied. Particularly, four beam suspensions are located at two sides of the mass aligned with edges of the mass, and the thickness of the beam is consistent with the thickness of the mass block in order to achieve multi-axis detection with high stability. The Mo/AlN/ScAlN/Mo composite films used as sensing elements are distributed on the surface of silicon-based cantilever beams. A theoretical model is first used to calculate the output characteristics. Meanwhile, the structural deformation, stress, and potential distribution of the proposed accelerometer are also simulated. Our experimental results show that the proposed accelerometer has great sensitivity and linearity under tri-axis direction accelerations.

## 2. Structural Design

**Figure 1:**
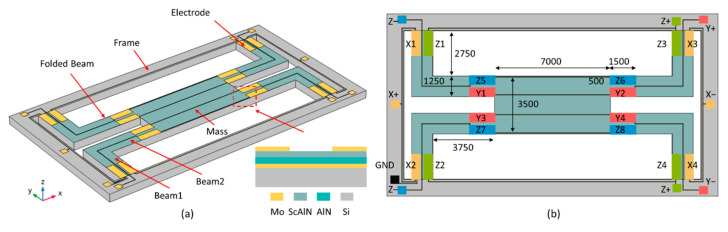
(**a**) Schematic view of the tri-axial piezoelectric accelerometer with folded beams; (**b**) Geometric parameter of the sensor and electrical connections of the electrode pads.

Figure 1a shows the schematic of the tri-axial accelerometer with a seismic mass suspended by four folded beams. Particularly, beam suspensions are located at two sides of the mass aligned with edges of the mass, and the thickness of the beam is consistent with the thickness of the mass block. The motivation of this design is to make the center of the mass and the center of the beam on the same plane. Moreover, the folded beams located at two sides of the mass can reduce the torsional deformation when measuring lateral acceleration compared with the traditional cross-shaped accelerometer. The geometric parameters of the piezoelectric accelerometer are listed in Table 1. Piezoelectric sensing elements consisted of Mo/AlN/ScAlN/Mo composite films are distributed on the surface of the silicon-based cantilever beams. The AlN thin film with tensile stress deposited underneath the ScAlN is used to compensate for the compressive stress of ScAlN thin film [24]. When the accelerometer is subjected to an external load, the entire structure will be deformed due to inertial force, and the maximum stress generally occurs at the fixed end of the beam. In addition, considering the complexity of the deformation of the tri-axial accelerometer, the sensitive elements need to be reasonably distributed at both ends of the beam to prevent signal superimposition and interference. In the design, the bottom electrode and piezoelectric layer are fabricated as a whole plate, while the top Mo electrodes are patterned into small rectangle electrodes placed at the end of the supported beams. A total of 16 top electrodes are segmented into Z_1_, Z_2_, Z_3_, Z_4_, Z_5_, Z_6_, Z_7_, Z_8_ (for *z*-axis sensing), Y_1_, Y_2_, Y_3_, Y_4_ (for *y*-axis sensing), X_1_, X_2_, X_3_, X_4_ (for *x*-axis sensing) (Figure 1b). The top electrodes Z_1_–Z_4_ (Z−) and Z_5_–Z_8_ (Z+) are respectively connected in parallel to detect the output signal when the structure is accelerated vertically (along the *z*-axis). When the structure is accelerated laterally (e.g., in Y-direction), the electrodes Y_1_, Y_2_ (Y+), and Y_3_, Y_4_ (Y−) at the position of the mass sides of beam2 are used. The same principles apply to the case in which the structure is accelerated laterally in X-direction, where electrodes X_1_, X_2_ (X+), and X_3_, X_4_ (Y−) are distributed on the frame sides of beam1. Moreover, the bottom electrode is designed as a floating potential.

## 3. Theoretical Model and Simulation

### 3.1. Theoretical Model

**Figure 2:**
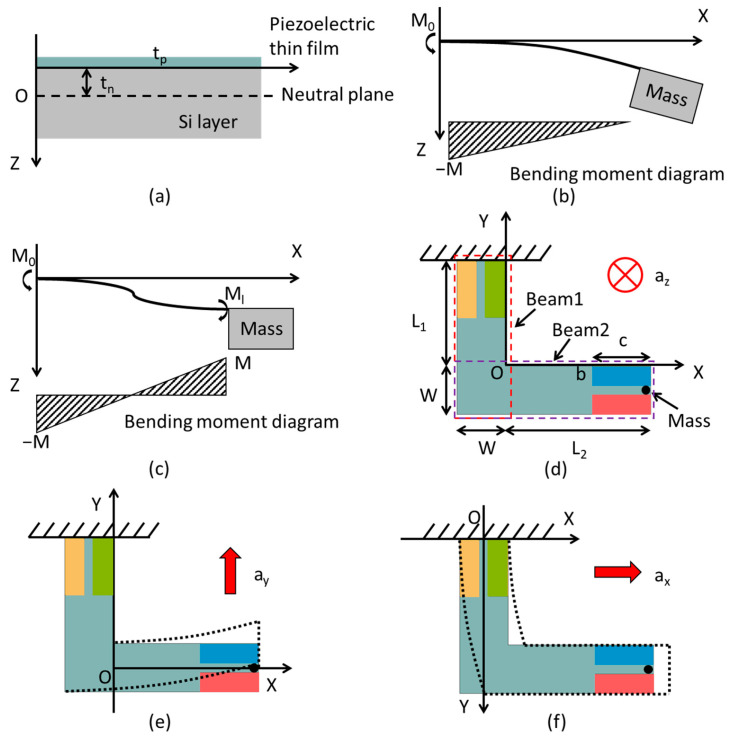
(**a**) Cross-sectional area of the suspended piezoelectric/silicon beam in the thickness direction of the device. Two deformations of the cantilever: (**b**) the direction of the bending moment of the beam is all same (Type 1); (**c**) the direction of the bending moments at both ends of the beam are opposite (Type 2). Schematic diagram of the tri-axial piezoelectric accelerometer when *z*-axis (**d**), *y*-axis (**e**), and *x*-axis (**f**) accelerations are applied respectively.

To evaluate the electric response of the tri-axial piezoelectric accelerometer, the sensitivities under *x*, *y*, *z*-axis direction accelerations are analyzed, respectively. According to the design, the model is a symmetrical structure, and the overall performance of the structure can be obtained by analyzing the 1/4 part. Moreover, beam1 and beam2 have different dimensions and boundary conditions, thus the sensors have different responses when *x* and *y*-axis accelerations are applied. When the fixed frame is subject to acceleration a, the inertial force of the structure induces a deflection of the cantilever beams, and we have the inertial force:

(1) $$F=\frac{1}{4}m_{0}a$$

where $m_{0}$ is the central mass. Since the thickness of the Mo electrodes is much smaller than the thickness of the substrate, the simplified model only contains the piezoelectric layer and the *Si* substrate (Figure 2a). The position *t_n_* of the neutral plane can be approximately calculated as:

(2) $$t_{n}=\frac{E_{Si}t_{Si}^{2}-E_{p}t_{p}^{2}}{2\left(E_{Si}t_{Si}+E_{p}t_{p}\right)}$$

where $E_{Si}$, $E_{p}$ and $t_{Si}$, $t_{p}$ are Young’s modulus and the thickness of the *Si* substrate and piezoelectric layer. The accelerometer’s charge sensitivity $S_{Q}$ and voltage sensitivity $S_{V}$ defined as:

(3) $$S_{Q}=\frac{Q}{a}=S_{V}C=\frac{VC}{a}$$

where *Q*, *V,* and *C* are output charge, open-circuit voltage, and capacitance, respectively.

When the structure is subjected to external forces, two forms of beam deformation occur. Type 1 is that the direction of the bending moment of the whole beam is the same (Figure 2b), and type 2 is that the direction of the bending moments at both ends of the beam is opposite (Figure 2c).

- Voltage sensitivity of *z*-axis

Figure 2d shows the schematic diagram of one of the folded beams. The upper end of beam1 is fixed, and the mass block is simplified as a point load at the end of beam2. Since the beams have the same thickness as the mass block, the effects of the beam1, beam2, and mass load should be considered at the same time. When the accelerometer is applied with *z*-axis load, beam1 shows type 1 deformation, and beam2 shows type 2 deformation. Considering the bending deformation caused by the weight of the beam itself, the bending moments of the beam1 ($M_{z1}\left(y\right)$) and beam2 ($M_{z2}\left(x\right)$) are as follows [17]:

(4) $$\begin{array}{cc} M_{z1}\left(y\right) & =M_{mass}+M_{beam2}+M_{beam1}\\ & =\frac{m_{0}a_{z}}{4}\left(y+W\right)+ma_{z}L_{2}\left(y+W\right)+ma_{z}\int_{-W}^{y}\left(y-y_{0}\right)dy_{0} \end{array}$$

(5) $$\begin{array}{cc} M_{z2}\left(x\right) & =M_{mass}-M_{beam2}\\ & =\frac{m_{0}a_{z}}{4}\left(\frac{L_{2}+W}{2}-x\right)-ma_{z}\int_{x}^{L_{2}}\left(x_{0}-x\right)dx_{0} \end{array}$$

where *m* is mass per unit length of beams, *W* is the width of the beam, *L*_1_ and *L*_2_ are the lengths of Beam1 and Beam2. The intensity of the induced electric field E in the piezoelectric film is:

(6) $$E=g_{31}E_{p}\frac{M}{WD}\left(t_{n}-z\right)$$

where $g_{31}$ is the piezoelectric voltage constant of piezoelectric material, and *D* is the bending modulus per unit width of the cantilever [25]. The open-circuit voltages of an electrode pad on beam1 and beam2 are calculated as:

(7) $$V_{z1}=\frac{1}{c}\int_{L_{1}-c}^{L_{1}}\int_{-t_{p}}^{0}E_{z1}\left(y,\;z\right)dzdy$$

(8) $$V_{z2}=\frac{1}{c}\int_{L_{2}-c}^{L_{2}}\int_{-t_{p}}^{0}E_{z2}\left(x,\;z\right)dzdx$$

where *c* is the length of the electrode pad. Finally, the *z*-axis signal can be obtained by subtracting the open-circuit voltage on the two electrodes:

(9) $$\begin{array}{cc} V_{z} & =V_{z1}-V_{z2}\\ & =\frac{g_{31}E_{p}a_{z}}{WD_{z}}\left(t_{n}t_{p}+\frac{t_{p}^{2}}{2}\right)\left[\frac{m_{0}}{8}\left(2L_{1}+L_{2}+W-2c\right)+mL_{2}\left(L_{1}+W-\frac{c}{2}\right)+\frac{m}{6}\left(3{\left(L_{1}+W\right)}^{2}+2c^{2}-3c\left(L_{1}+W\right)\right)\right] \end{array}$$

- Voltage sensitivity of *y*-axis

When the *y*-axis acceleration is applied, despite there exist shear stress during deformation, the corresponding piezoelectric coefficient is zero. Also, compare to beam2, the deformation of beam1 is so small that it can be ignored. To simplify the model, beam1 is assumed to be a rigid body remaining stationary and beam2 undergoes pure bending deformation, as shown in Figure 2c. In this case, beam2 shows the type 2 deformation and the bending moment is:

(10) $$\begin{array}{cc} M_{y} & =M_{mass}-M_{beam2}\\ & =\frac{m_{0}a_{y}}{4}\left(\frac{L_{2}+W}{2}-x\right)-ma_{y}\int_{x}^{L_{2}}\left(x_{0}-x\right)dx_{0} \end{array}$$

Because the bending is along the *y*-axis, and the piezoelectric thin film is on the vertical plane of the bending direction, the neutral axis is the center axis of the silicon-based beam2 along the *x*-axis direction. The terminals Y+ and Y− are located on both sides of the neutral plane, the opposite strain will cause opposite potentials, and the output voltage is:

(11) $$\begin{array}{cc} V_{y} & =-\frac{2}{c}\int_{L_{2}-c}^{L_{2}}\int_{-\frac{W}{2}}^{b-\frac{W}{2}}g_{31}E_{p}\frac{M_{y}}{\left(t_{Si}+t_{p}\right)D_{y}}ydydx\\ & \approx \frac{g_{31}E_{p}a_{y}\left(bW-b^{2}\right)}{t_{Si}D_{y}}\left[\frac{m_{0}}{8}\left(c+W-L_{2}\right)-\frac{mc^{2}}{6}\right] \end{array}$$

- Voltage sensitivity of *x*-axis

Similarly, when *x*-axis acceleration is applied, the deformation of beam2 is negligible, and it is assumed to be a rigid body as a part of the mass block. The neutral axis is the center axis of the silicon-based beam1 along the *y*-axis direction. Beam1 shows the type 2 deformation and the bending moment is:

(12) $$\begin{array}{cc} M_{x} & =M_{beam2+mass}+M_{beam1}\\ & =\left(\frac{m_{0}}{4}+mL_{2}\right)a_{x}\left(\frac{L_{1}+W}{2}-y\right)+ma_{x}\int_{y}^{L_{1}+W}\left(y-y_{0}\right)dy_{0} \end{array}$$

The terminals X+ and X− have opposite potentials, and the output voltage can be calculated as:

(13) $$\begin{array}{cc} V_{x} & =-\frac{2}{c}\int_{0}^{c}\int_{-\frac{W}{2}}^{b-\frac{W}{2}}g_{31}E_{p}\frac{M_{x}}{\left(t_{Si}+t_{p}\right)D_{x}}xdxdy\\ & \approx \frac{g_{31}E_{p}a_{x}\left(bW-b^{2}\right)}{t_{Si}D_{x}}\left[\left(\frac{m_{0}}{4}+mL_{2}\right)\frac{L_{1}+W-c}{2}+\frac{m\left(c^{2}-3c\left(L_{1}+W\right)+3{\left(L_{1}+W\right)}^{2}\right)}{6}\right] \end{array}$$

Since the parameter D satisfies $D_{z}<D_{y}=D_{x}$, combined with the data in Table 1 and Table 2, the results from equations from (9), (11), and (13) show $|V_{z}|>\left|V_{x}\right|>\left|V_{y}\right|$ under the same amplitude acceleration. Using Equation (4), the charge sensitivity and voltage sensitivity of the tri-axial accelerometer can be calculated.

### 3.2. Simulation

**Figure 3:**
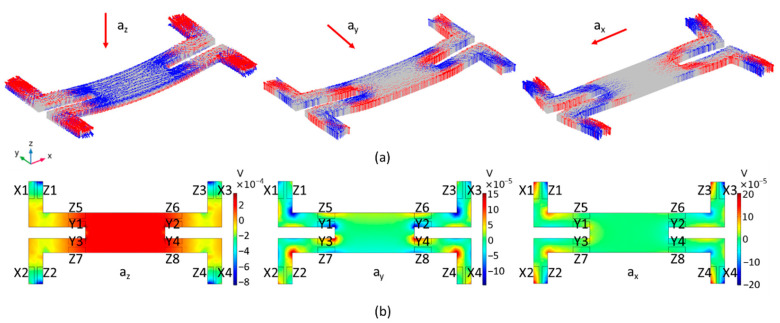
Numerical simulation results of the stationary analysis using COMSOL. (**a**) The structural deformation and stress distribution along the *x*, *y*, *z*-axis: the red arrow indicates tensile stress and the blue arrow indicates compressive stress; (**b**) The voltage distribution of tri-axial piezoelectric accelerometer along *x*, *y*, *z*-axis at the acceleration level of 1 g.

To estimate the structural deformation, stress, and potential distribution of the suspension structures along the *x*, *y*, *z*-axis, respectively, stationary analysis of finite element analysis (FEA) was conducted. As shown in Figure 3a, when a *z*-axis acceleration is applied to the structure, the upper surface of beam1 is all tensile stress while the two ends of beam2 show opposite stress. When a *y*-axis acceleration is applied to the structure, the sides at both ends of beam2 show opposite stress. Similarly, the sides at both ends of beam1 show opposite stress when an *x*-axis acceleration is applied. The simulation results of deformation and stress verify the deformation types in the previous section.

**Figure 4:**
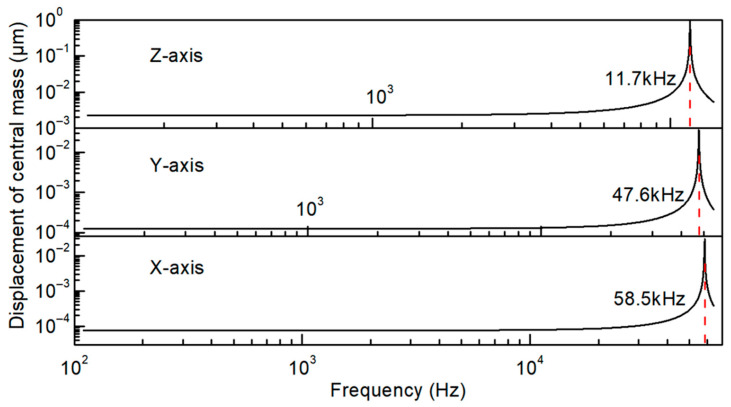
The frequency response of the tri-axial accelerometer: displacement vs. frequency.

It can be seen from Figure 3a that the four silicon beams experience the same bending deformation under the *z*-axis acceleration, but exhibit symmetric deformation under the acceleration of the *x* and *y*-axis. When *z*-axis acceleration is applied, opposite stresses are generated at the position of the frame sides of beam1 and the mass sides of beam2. As a consequence, there exists a finite voltage between the parallel-connected Z_1_–Z_4_ (Z−) and Z_5_–Z_8_ (Z+), as shown in Figure 3b. When *y*-axis acceleration is applied, opposite stresses occur on the upper and lower sides of beam2 near the mass, thus there will be a potential difference between the electrodes Y_1_, Y_2_ (Y−), and Y_3_, Y_4_ (Y+). Since there is a corner at the junction between the beam2 and the mass, this leads to stress concentration and improved potential. Similarly, when *x*-axis acceleration is applied, opposite stresses occur on the left and right sides of beam1 near the frame, thus there will be a potential difference between the electrodes X_1_, X_2_ (X+), and X_3_, X_4_ (X−). According to the stationary analysis of simulation, when the acceleration of the same amplitude along the *x*, *y*, *z*-axis is applied to the accelerometer, the output along *z*-axis is the highest, and the output along *y*-axis is the lowest, which is consistent with the theoretical analysis. The frequency domain analysis describes the response of the accelerometer at different frequencies. The resonance frequencies of the tri-axial accelerometer along the *x*, *y*, and *z*-axes are 58.5 kHz, 47.6 kHz, and 11.7 kHz, respectively. Moreover, the response of the accelerometer below 1 kHz is linear and stable (Figure 4).

## 4. Fabrication and Measurement

**Figure 5:**
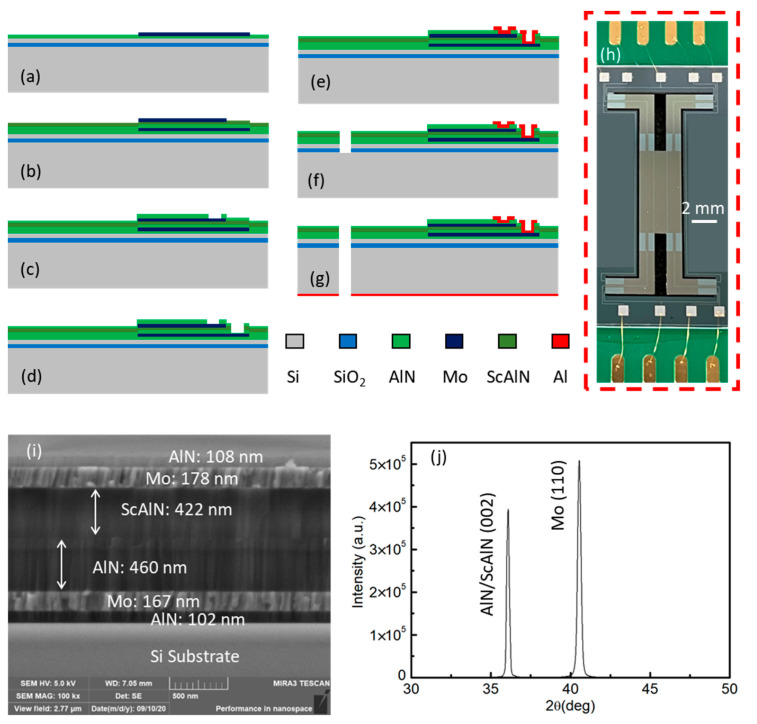
The main fabrication process of the chip: (**a**) bottom electrode patterning; (**b**) top electrode patterning; (**c**) top opening; (**d**) bottom opening; (**e**) Al pad patterning; (**f**) top releasing; (**g**) bottom releasing; (**h**) Front-side photo of a complete tri-axial piezoelectric accelerometer; (**i**) The SEM image of a cross-section of a 2 micron length of the piezoelectric stack of the accelerometer; (**j**) The XRD spectrum of the thin films.

The piezoelectric accelerometers consisted of four silicon cantilever beams ended with a mass block are produced by standard micromachining technology. The process flow of the accelerometer with Mo/AlN/ScAlN/Mo sensing layers is schematically shown in Figure 5a–g. To start, a 6-in SOI wafer comprised of a 500 μm thick handle layer, 1 μm thick buried oxide layer, and 50 μm device layers is used. The next step is the deposition of a Mo (200 nm) bottom electrode with an AlN (100 nm) seed layer, followed by the patterning of the bottom electrode (Figure 5a). The AlN (500 nm) and ScAlN (500 nm) films were deposited by reactive sputtering in order. The Mo (200 nm) top electrode deposition and patterning finish the piezoelectric sandwich stack (Figure 5b). Then, thin AlN films (100 nm) were deposited as a passive layer. The top electrode and bottom electrode were exposed by etching the square holes, respectively (Figure 5c,d). Electrode pads were formed by depositing and patterning metal Al (1 μm) (Figure 5e). Next, the trench patterning is done, which defines the cantilever shape (Figure 5f). The etched layers include a piezoelectric layer (AlN, ScAlN), Si substrate, and oxide layer. On the backside of the wafer, the metal Al (300 nm) is deposited and patterned as a hard mask for Deep reactive ion etching (DRIE) (Figure 5g). Bosch process is used to release the mass and cantilevers.

Figure 5i shows the scanning electron microscope (SEM) image of the fabricated device. However, the thickness of the thin films is smaller than the designed value due to the deviation of the fabrication process. Figure 5j shows the Measured X-ray diffraction (XRD) spectrum of the Mo/AlN/ScAlN/Mo composite thin film. Since the (002) peaks of AlN and ScAlN are very close and superimposed, the curve just shows two sharp peaks corresponding to AlN/ScAlN (002) and Mo (110).

The device is bonded on a printed circuit board (PCB), which has a cavity under the die to ensure the free movement of the structure in the *z*-axis direction. The electrode pads on the die are wire-bonded to the gold pads on the PCB for connecting the subsequent conditioning amplifier. Figure 5h shows the images of the fabricated MEMS piezoelectric accelerometer fixed on a PCB.

**Figure 6:**
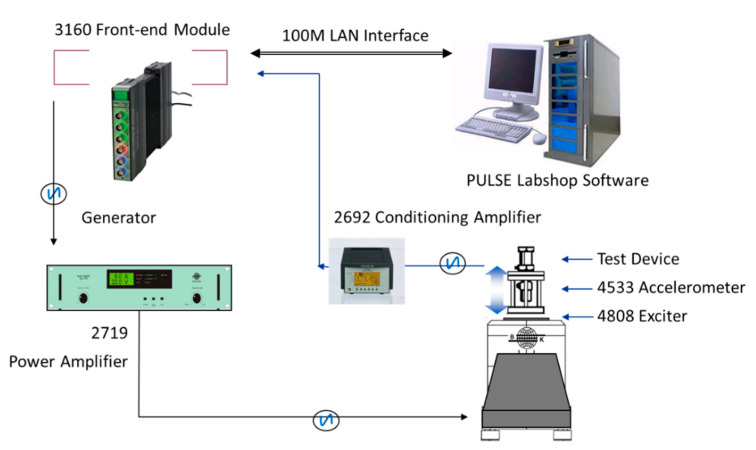
Experimental setup for output measurements.

The fabricated piezoelectric accelerometers are measured using a setup illustrated in Figure 6. The vibration test calibration system can be used for measuring charge sensitivity characteristics over a frequency range from 3 Hz to 6.4 kHz. The PCB is rigidly fixed on the calibration fixture of the exciter (B&K 4808), while a reference accelerometer (B&K 4533-B) is installed under the fixture to measure the real-time acceleration generated by the exciter. The amplitude, frequency, and bandwidth of sinusoidal vibration can be controlled by B&K PULSE Labshop software and a power amplifier (B&K 2719). The output charge signal from the piezoelectric stack is converted into a voltage signal by a conditioning amplifier (NEXUS 2692) and then read by the data acquisition module of the frond-end setup (B&K 3160). By changing the fixture and clamping position, the testing system can measure the sensitivity in the *x*, *y*, *z*-axis directions, respectively. In addition, the capacitances of terminal X, Y, Z ($C_{x}=124pF,\;C_{y}=133pF,\;C_{z}=278pF$) are measured by LCR Meter (E4980A), respectively.

## 5. Results

**Figure 7:**
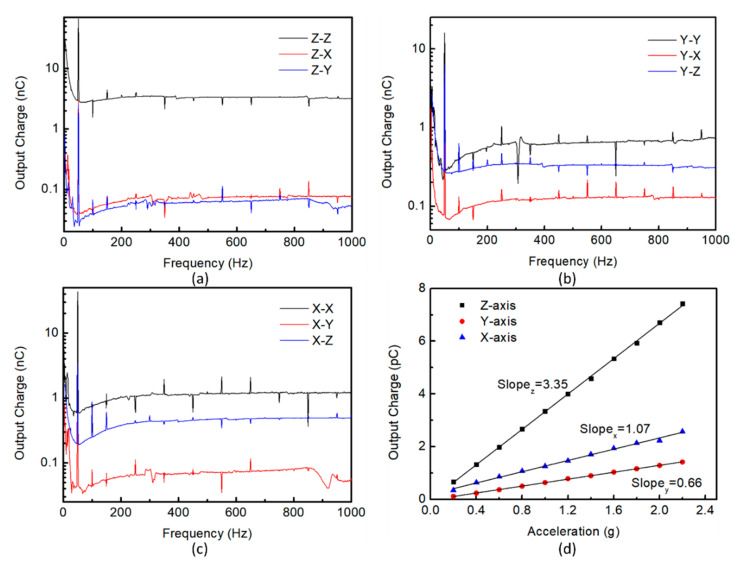
Output charge vs. frequency under 1g acceleration in the frequency range of 3 Hz–1 kHz: (**a**) Z–Z (Terminal Z/*z*-axis acceleration), Z–X (Terminal Z/*x*-axis acceleration), Z–Y (Terminal Z/*y*-axis acceleration) (**b**) Y–Y (Terminal Y/*y*-axis acceleration), Y–X (Terminal Y/*x*-axis acceleration), Y–Z (Terminal Y/*z*-axis acceleration) (**c**) X–X (Terminal X/*x*-axis acceleration), X–Y (Terminal X/*y*-axis acceleration), X–Z (Terminal X/*z*-axis acceleration); (**d**) The output charge of the accelerometer under different acceleration at the frequency of 500 Hz.

The sensitivity, linearity, and operating frequency range are important parameters to evaluate the performance of a tri-axial piezoelectric accelerometer. The frequency spectra for the tri-axial accelerometer along the *x*, *y*, *z*-axis is measured under 1g acceleration over a frequency range from 3 Hz to 1 kHz, as shown in Figure 7a–c. The error below 50 Hz is the systematic error of the whole measurement, and the noise over 50 Hz–1 kHz is mainly caused by 50 Hz harmonic interference. For the tri-axial accelerometer operating under *z*-axis acceleration, the amplitude rises in the frequency of 52 Hz–1 kHz is about 10.3% (<12% or 1dB [29]). However, the frequency response along the *x* and *y*-axis is poor compared with *z*-axis at low frequencies, and the amplitude rises about twice in the frequency of 52 Hz–500 Hz. The 500–900 Hz frequency range is a relatively stable working range for *x*, *y*, *z*-axis detection. To evaluate the effect of the lateral acceleration, the corresponding responses in the other two directions are also measured. Obviously, when measuring the output of terminal Z, the output charge caused by *x* and *y*-axis acceleration is significantly lower than *z*-axis, indicating the low transverse effect (Figure 7a). When measuring the response in the Y direction, the acceleration of the *z*-axis produces higher interference than the acceleration of the *x*-axis (Figure 7b). The test response in the *x*-axis direction also has the same trend (Figure 7c). One of the reasons is that due to the process errors, the structure of the accelerometer is not completely symmetrical. The stress generated by the accelerometer under the acceleration in the Z direction is greater than the stress generated by the acceleration in the X and Y directions. Once the asymmetric deformation of the device occurs, the interference of the load in the *z*-axis direction on the *x* and *y*-axis detection will appear. Based on the simulation results in Figure 3, although lateral acceleration will produce stress and potential, the symmetrical potential distribution and designed connections of sensing elements can offset the electric potential difference caused by loads in other directions. If the symmetry of the structure and the stability of the test environment can be ensured, the tri-axial accelerometer can have lower lateral coupling. The output charge characteristics of the tri-axial accelerometers are measured by applying 0.2–2.2 g acceleration (0.2 g increment) along the *x*, *y*, *z*-axis direction of the device at a frequency of 500 Hz. As shown in Figure 7d, the output characteristics of the accelerometer are approximately all linear. With the increase of applied acceleration, accumulated charge increase from 0.354 pC, 0.117 pC, and 0.667 pC to 2.577 pC, 1.42 pC, and 7.426pC, respectively. The slopes of fitting lines represent the charge sensitivity of the tri-axial accelerometer and the measured $S_{Q,X}$, $S_{Q,Y}$, and $S_{Q,Z}$ are ~1.07 pC/g, ~0.66 pC/g, and ~3.35 pC/g, respectively.

## 6. Conclusions

In this paper, a tri-axial piezoelectric accelerometer with folded beams has been designed, fabricated, and tested for vibration detection. Since the thickness of folded beams is the same as the thickness of mass block, and it is supported symmetrically at both sides, the accelerometer can work stably over a large frequency range. Compared with the traditional cross-shaped accelerometer, the deformation of the thick folded beams can be simplified as pure bending under tri-axial direction accelerations. A total of 16 sensing elements are distributed at the end of the beams. FEA simulation is performed to analyze the deformation and output characteristics under the *x*, *y*, and *z*-axes accelerations. The results calculated from the analytical model and simulation show that the output characteristics satisfy $\left|V_{z}\right|>\left|V_{x}\right|>\left|V_{y}\right|$. Micromachining technology is used to fabricate the tri-axial piezoelectric accelerometer. The experimental results show that the charge sensitivity along the *x*, *y*, and *z*-axis could reach up to ~1.07 pC/g, ~0.66 pC/g, and ~3.35 pC/g. Further challenges will still remain to design tri-axial piezoelectric MEMS accelerometers. The proposed piezoelectric accelerometer with folded thick beams has the potential to detect tri-axial vibration acceleration with great sensitivity and linearity.

## Figures and Tables

**Table 1:** The structure parameters of the tri-axial MEMS accelerometer.

Parameters	Dimensions (μm)
Length of the mass	7000
Width of the mass	3500
Length of the beam1	2750
Width of the beam1	1250
Length of the beam2	3750
Width of the beam2	1250
Length of the top electrode pad	1500
Width of the top electrode pad	500
The thickness of the Si substrate	550

**Table 2:** Main parameters of the materials [26,27,28].

Material	Density (kg/m³)	Young’s Modulus (GPa)	Piezoelectric Coefficient *d*_31_ = *g*_31_*ε*_r_*ε*_0_ (pC/N)	Relative Permittivity *ε*_r_
AlN	3300	244	−1.784	9
Sc_0.2_Al_0.8_N	3587	189	−3.436	13.7
Si	2320	160	-	-

## Data Availability

Data is contained within the article.
